# Neural representations of naturalistic person identities while watching a feature film

**DOI:** 10.1162/imag_a_00009

**Published:** 2023-08-21

**Authors:** Clare Lally, Nadine Lavan, Lucia Garrido, Maria Tsantani, Carolyn McGettigan

**Affiliations:** University College London, London, United Kingdom; Department of Psychology, Queen Mary University of London, London, United Kingdom; City, University of London, London, United Kingdom; Birkbeck, University of London, London, United Kingdom

**Keywords:** person perception, voice recognition, face recognition, fMRI, representational similarity analysis, naturalistic, task-free

## Abstract

Recognising other people in naturalistic settings relies on differentiating between individuals (“telling apart”), as well as generalising across within-person variability (“telling together”; Burton, 2013; Lavan, Burston, & Garrido, 2019; Lavan, Burton, et al., 2019). However, previous neuroscientific investigations of face and voice recognition have tended to measure identity-related responses and representations using tightly controlled stimuli, thus under sampling the naturalistic variability encountered in everyday life. In this study, we tested whether cortical regions previously implicated in processing faces and voices represent identities during naturalistic and task-free stimulation. Representational similarity analyses were conducted on functional MRI datasets collected while human participants watched feature-length movies. Identity representations—defined as similar response patterns to variable instances of the same person (“telling together”), and dissimilar patterns in response to different people (“telling apart”)—were observed in established face and voice processing areas, across two independent participant groups viewing different sets of identities. We also explored contributions of face versus voice information to identity representations, finding more widespread preferential sensitivity to faces. We thus characterise how the brain represents identities in the real world, for the first-time accounting for both “telling people together” and “telling people apart.” Despite substantial differences to previous experimental research, our findings align with previous work, showing that similar brain areas are engaged in the representation of identities under experimental and naturalistic exposure.

## 1 Introduction

The ability to recognise and individuate other people is essential for navigating everyday life. When attending a social gathering, we can determine whether we have met someone before and act accordingly: Do we greet them in a familiar manner or do we need to introduce ourselves? Similarly, when watching a movie, we need to process the characters’ identities to follow the plot. While information about a person’s identity can be gleaned from a number of sources, the most salient signals are arguably people’s faces and voices. These signals naturally vary, as people look and sound different from moment to moment depending on a number of contextual factors (e.g., for the face: head angle, lighting, facial expression; for the voice: room acoustics, social context, state of arousal). Successful recognition therefore requires people to not only “tell apart” different identities, but also “tell together” multiple instances of the same person into a single perceived identity (Burton, 2013; Lavan, Burston, & Garrido, 2019; Lavan, Burton, et al., 2019). To support this, people may rely on stable representations of identities to overcome variability during identification (Belin et al., 2011; Bruce & Young, 1986; Kreiman & Sidtis, 2011; Lavan, Burston, & Garrido, 2019; Lavan, Burton, et al., 2019; Maguinness et al., 2018; Stevenage & Neil, 2014). The ability to generalise is incorporated into existing hierarchical models of face and voice processing (Belin et al., 2004; Bruce & Young, 1986; Haxby et al., 2000; Maguinness et al., 2018), which propose that people initially analyse visual/auditory features, before comparing them to stored knowledge, or representations, of familiar faces/voices. In these models, recognition of an identity via stored knowledge then facilitates access to higher-order information, such as person identity and associated biographical information. Thus, forming a stable representation of somebody’s identity may be a fundamental prerequisite for consistently accurate person identification.

A large body of past research has identified brain regions that reliably respond to face and voice stimuli, and provides supporting evidence for the processes proposed by theoretical models. Neuroimaging studies have localised distinct face- and voice-processing networks (for faces, see Pitcher et al., 2011, Rossion et al., 2012; for voices, see Luthra, 2021, Maguinness et al., 2018), in which different areas appear to engage in various hierarchical stages of low-level structural analysis and higher-order identity processing. Face-selective regions include regions within the lateral occipital cortex and right fusiform gyrus, referred to as the occipital face area (OFA) and fusiform face area (FFA) respectively (Kanwisher et al., 1997; Pitcher et al., 2011). Voice-selective regions, often called the temporal voice areas, have been localised to bilateral superior temporal cortices (STG/STS; Belin et al., 2004). Within these temporal voice areas, both posterior and anterior regions in STG and STS have been associated with voice identity processing (Andics et al., 2010; Belin & Zatorre, 2003; Luthra, 2021; Maguinness et al., 2018; Schall et al., 2014; von Kriegstein & Giraud, 2004; von Kriegstein et al., 2003, 2010; Zäske et al., 2017). Researchers have also identified brain regions that support multimodal person perception, in which face and voice information is thought to be integrated or exchanged. These regions include the right posterior STS (Anzellotti & Caramazza, 2017; Campanella & Belin, 2007; Davies-Thompson et al., 2019; Deen et al., 2015; Tsantani et al., 2019; Watson, Latinus, Charest, et al., 2014; Watson, Latinus, Noguchi, et al., 2014), and midline regions including the precuneus, posterior cingulate, frontal pole, and orbitofrontal cortex (Shah et al., 2001).

Within these core face-, voice-, and person-selective brain regions, further work has investigated distinct neural response patterns to different identities *in relation to one another*. These enquiries have been advanced by the advent of multivariate pattern analysis techniques, which compare voxel-wise spatial patterns of activity. Researchers have detected distinct neural response patterns to different identities in the anterior temporal lobe (Anzellotti & Caramazza, 2016; Anzellotti et al., 2014; Kriegeskorte et al., 2007), occipitotemporal cortex (Anzellotti et al., 2014), and right anterior fusiform gyrus (Nestor et al., 2011) for face stimuli; and bilateral superior temporal sulci, primary auditory cortex, and middle temporal gyrus (Formisano et al., 2008; Hasan et al., 2016) for voice stimuli. Some studies have also incorporated investigations into tolerance to within-person variability (telling together), by identifying regions capable of *image-invariant* face identity decoding. Regions including the right anterior temporal lobe (Anzellotti & Caramazza, 2016; Anzellotti et al., 2014), occipitotemporal cortex (Anzellotti et al., 2014), and right anterior fusiform gyrus (Nestor et al., 2011) have demonstrated similar patterns of activity in response to variable exemplars of the same face identity, despite visual differences such as image rotation, partial obscuration, or emotional expression. For voices, Formisano et al. (2008) demonstrated that speaker identity can be decoded from listeners’ activation patterns in the right STS and primary auditory cortex, in which activation patterns differentiated between speaker identities but showed consistency across speech content (different vowel sounds). Finally, recent work has investigated whether neural identity representations extend beyond single modalities: Tsantani et al. (2019) observed stimulus-invariant discrimination of face identities in OFA and FFA, and of voice identities in bilateral STG/STS. Cross-modal identity decoding was detected in the right posterior STS, which discriminated between different face identities based on neural responses to voice identities, and vice versa (see also Hasan et al., 2016, for similar findings).

Existing research has advanced our understanding of how the brain processes identity information. However, one prevalent limitation within the field is that most studies have used carefully designed experiments that inevitably fail to capture aspects of identity processing in everyday life. Participants are typically presented with brief context-free exposures, where stimuli are highly controlled (e.g., passport-style portraits; sustained vowels) and restricted to one or few exemplars. Further, participants are frequently required to perform specific tasks (e.g., button press responses), which consequently create artificial settings for identity processing. Recent developments in other cognitive neuroscience domains have seen increased use of task-free, naturalistic stimulation to investigate the neurobiology underpinning cognitive processes (Eickhoff et al., 2020; Hamilton & Huth, 2020; Hasson et al., 2008). Naturalistic in-scanner experiences produce greater test-retest reliability compared with task-based or resting-state fMRI, which may be not only due to greater participant compliance (Madan, 2018; Vanderwal et al., 2015) but also due to eliciting neural activity that is more reflective of everyday mental operations (Vanderwal et al., 2017; Wang et al., 2017). Further, the ability to use extended exposure and richer context presents powerful opportunities to study how the brain tracks and encodes information across hierarchical levels (see Huth et al., 2016; Wehbe et al., 2014; Hamilton & Huth, 2020 for examples in language research). Thus, task-free naturalistic stimulation has the potential to provide unique insights into the neural underpinnings of identity processing, with advantages that cannot be achieved with traditional experimental paradigms. Neuroimaging data from participants watching feature-length films can be leveraged to allow for ecologically valid investigation of neural identity representations: Multiple characters are encountered over hours of audio-visual footage rather than in brief exposures; face and voice stimuli are dynamic, naturalistic, and capture within-person variability across utterances and scenes; presentations of individual identities are contextualised within an ongoing narrative; and participants are actively engaged with the identities in the absence of laboratory-style task demands.

In the current study, we investigated whether person identities encountered during naturalistic, task-free stimulation are represented in established face-, voice-, and person-selective regions of the brain. To achieve this, we analysed open-access MRI datasets, in which participants watched feature-length movies (Aliko et al., 2020). We used representational similarity analysis, a multivariate approach based on the premise that stimuli that share representational qualities will evoke similar neural response patterns in the relevant regions that are sensitive to this type of information (Kriegeskorte et al., 2008). As a theoretical basis, we predicted that brain areas representing person identity should produce divergent patterns in response to different identities (“telling apart”), but show relative consistency in response to variable exposure to the same identity (“telling together”; Burton, 2013; Lavan, Burston, & Garrido, 2019; Lavan, Burton, et al., 2019). We expected to find such representations in regions previously reported to process face, voice, and multimodal person identities (Belin et al., 2004; Kanwisher et al., 1997; Maguinness et al., 2018; Shah et al., 2001; Tsantani et al., 2019). Our first two analyses of these MRI data identified brain representations of person identities based on context-rich, ecologically valid stimulation. These analyses were conducted on two independent datasets, in which participants watched either the documentary *Citizenfour* (Analysis 1) or the romantic comedy *500 Days of Summer* (Analysis 2). We observed internal replication across two separate participant groups, who were exposed to different identities in different feature-length movies. In light of the multimodal nature of how people are encountered while watching a feature film, the final exploratory analysis (Analysis 3) aimed to dissociate the contributions of face versus voice information to the neural representations of identity.

## 2 Analysis 1: *Citizenfour*

Our first aim was to uncover brain areas that are sensitive to person identity in naturalistic audio-visual stimuli. We analysed the data of 18 participants who watched the documentary *Citizenfour* during continuous functional MRI data acquisition, and assessed the similarity of neural responses to different identities (“telling apart”), as well as across multiple variable instances of the same identity (“telling together”).

### 2.1 Materials and methods

#### 2.1.1 MRI data

We analysed data from the Naturalistic Neuroimaging Database (Aliko et al., 2020), obtained from 18 participants (nine female, aged 19-58 years old, mean age: 27 years) who watched the documentary film *Citizenfour*. All participants were right-handed native English speakers, with no known history of neurological disorders and with unimpaired hearing and vision. The MRI data were acquired on a 1.5 T Siemens MAGNETOM Avanto scanner with a 32-channel head coil (Siemens Healthcare, Erlangen, Germany). Functional data were acquired using a multiband EPI sequence (TR = 1 second, TE = 54.8 ms, flip angle of 75°, 40 interleaved slices, resolution = 3.2 mm isotropic), with 4x multiband factor and no in-plane acceleration. Each participant also underwent a high-resolution T1-weighted MPRAGE anatomical MRI scan (TR = 2.73 seconds, TE = 3.57 ms, 176 sagittal slices, resolution = 1.0 mm). Prior to analyses, the data had been pre-processed by Aliko et al. (2020). Pre-processing included motion correction, slice time correction, and co-registration of the functional images to the T1-weighted anatomical image. Both functional and anatomical scans were aligned to the Montreal Neurological Institute template brain. We opted to use pre-processed data that had foregone spatial smoothing, as differences in adjacent voxels may provide valuable information for pattern analyses (Kriegeskorte et al., 2008). Further details for acquisition and pre-processing procedures are published in Aliko et al. (2020).

### 2.2 Experimental design and statistical analysis

#### 2.2.1 Representational similarity analysis

Representational similarity analysis compares the structure or geometry of representations across different types of data or models (Kriegeskorte et al., 2008). We compared similarities in neural response patterns to multiple audio-visual stimuli, depicting specific individuals. These comparisons (expressing observed neural dissimilarity) were then compared with a hypothetical model, which expressed that within-identity comparisons should show similar patterns of neural activity, and across-identity comparisons should show dissimilar patterns of neural activity. Our aim was to probe neural representations that were sensitive to identity but stable across variable stimuli of the same person, therefore we pre-specified events of interest from the documentary *Citizenfour* that would best enable us to draw same and different identity comparisons. We identified the two people who appeared most prominently in the film (Edward Snowden and Glenn Greenwald), noted scenes in which each person appeared, and isolated time events for instances of each person’s speech during which their face was visible on-screen throughout. Scenes were defined based on a change of setting and/or time point within the narrative of the feature film, and labelled based on the order that they appeared within the film (i.e., interview in Edward Snowden’s hotel room, meeting at a data security summit). An utterance was defined as continuous speech from one person for a duration of 2 seconds or more, without pauses or overlapping speech from another talker. During these events, the speaker’s face was the only face on-screen. There were no restrictions on the appearance of the face (i.e., whether the speaker was front or side-facing, or whether it was only the speaker’s face or their full body in view). Finally, we filtered our time points of interest to only include utterances from scenes in which the person had a total speech duration of at least 4.5 seconds. Total speech duration was not necessarily based on 4.5 seconds of continuous speech: In the majority of cases, this was formed from multiple utterances, which lasted 2 seconds or longer. The procedure described above resulted in a final list of scenes with substantial dialogue for each identity. Each of these identity-scene combinations are henceforth referred to as items, composed of multiple instances of audio-visual speech uttered by a single specific person within a given scene. There were 12 items based on 94 utterances of Edward Snowden’s speech (1192 seconds in total) and 10 items based on 47 utterances of Glenn Greenwald’s speech (411 seconds in total). Examples of the modelled audio-visual instances of identities can be found in the online supplementary materials on the Open Science Framework (https://osf.io/vu23z/).

We then used these items to construct a model representational dissimilarity matrix (RDM), which expressed our predictions on the expected similarity of neural responses based on person identity (see Fig. 2A). This RDM included within-identity comparisons (e.g., *EDWARD 1—EDWARD 2*) and across-identity comparisons (e.g., *EDWARD 1—GLENN 1).* Within-identity comparisons were coded as similar (0; conceptually indexing “telling together”), whereas across-identity comparisons were coded as dissimilar (1; conceptually indexing “telling apart”). As the RDM was symmetrical, only the lower diagonal half of the matrix was included within the analysis. This ensured that item comparisons were not duplicated, and it prevented items from being compared with themselves.

We then constructed observed neural dissimilarity matrices, which compared actual dissimilarity between neural responses to our matrix items at each voxel. We used *SPM12* (Friston et al., 1994) to construct a general linear model, which included separate regressors for each of the items in our RDMs. Hence, each regressor was modelled based on multiple events of an individual person’s speech within a single scene; events within each regressor were modelled based on single utterances, from the onset of the first word to the offset of the last word within a sequence of uninterrupted speech without pauses. Timings were derived from time-stamped transcriptions of individual spoken words, which were automatically annotated by Aliko et al. (2020) using the machine-learning speech-to-text transcription tool “Amazon Transcribe” (Amazon World Services; https://aws.amazon.com/transcribe/). Event durations were modelled in seconds, and convolved with the canonical hemodynamic response function. After model estimation, we created contrast estimate maps for each item (identity-scene combination) versus baseline. The baseline was modelled from 100 events in the movie when there were no visible faces or audible speech (total duration: 9 minutes 16 seconds). We thus obtained brain responses to multiple instances of audio-visual speech of two different identities across various different scenes for each participant. Observed neural dissimilarity between items was calculated at voxel level by comparing the neural response pattern across RDM items. Pearson correlations were used to compute similarity in neural response patterns between items, and this was subtracted from one to express dissimilarity. This resulted in an observed neural dissimilarity matrix for each voxel location, which demonstrated how dissimilar neural patterns of activation were between utterances produced by different people and/or in different scenes. This procedure was repeated for each individual participant.

**Fig. 1.**
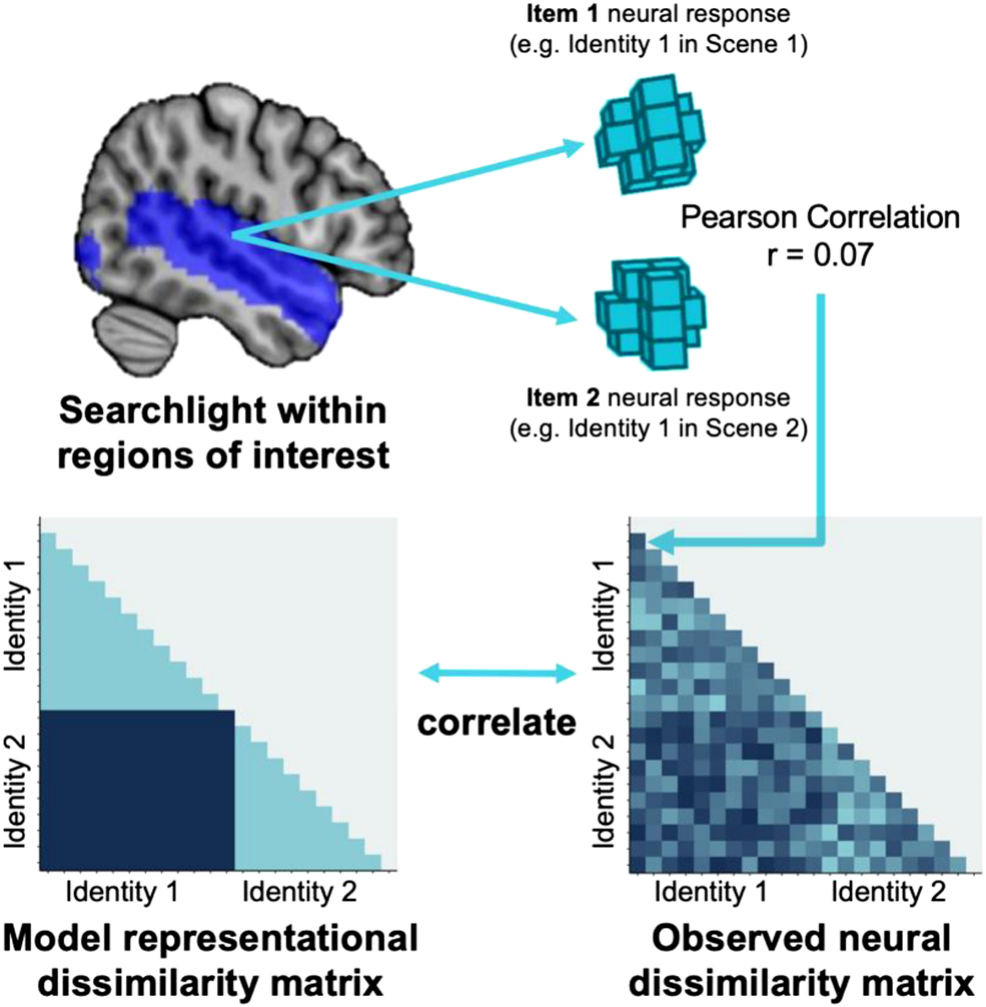
Illustration of the representational similarity analysis procedure, conducted on participant-level data using a searchlight approach within a predefined searchlight mask. In this study, an item corresponds to a unique identity-scene combination (see text for full description of the procedure).

Voxel-wise observed neural dissimilarity matrices were compared to the model RDM of person identity using a representational similarity analysis searchlight approach. To conduct these analyses, we used the CoSMo MVPA toolbox (Oosterhof et al., 2016) in MATLAB (MATLAB_R2020b, 2020). The full procedure is illustrated in Figure 1A. Searchlight analyses were conducted for each participant, whereby a spherical searchlight was used to extract voxel-wise neural response patterns for each item included in the RDM. Searchlight analyses were conducted within a predefined mask, based on regions of interest identified in a separate study (outlined further below). Neural response patterns were systematically recorded for each item at the central voxel of each searchlight based on patterns of activity in the surrounding 100 voxels. The response pattern for each item was compared to other items and used to construct the observed neural dissimilarity matrix for a given searchlight location. For each voxel within the searchlight mask, the observed neural dissimilarity matrices were then compared to the identity model RDM using Pearson correlation coefficients. These correlation values were then Fisher-to-*z*-transformed to create a normalised distribution and enable later comparisons across participants. For each participant, this procedure resulted in a brain map of Fisher-transformed correlation values for each voxel within the searchlight mask. Correlation values expressed how well the identity RDM characterised the observed neural dissimilarity in response to speech across different identities and/or scenes. Thus, higher correlation values indicate neural representations that are sensitive to person identity.

Finally, correlation maps were analysed at group level by conducting voxel-wise one-sample t-tests to determine whether correlations significantly differed from zero. This produced a group-level brain activation map of corresponding *z*-scores. Statistics were adjusted for multiple comparisons using threshold-free cluster enhancement (TFCE; Smith & Nichols, 2009). The TFCE procedure was carried out as follows: Voxel-wise values represented the amount of cluster-like local spatial support while controlling family-wise error rate, calculated based on the spatial extent (mass) and height (peak) of activation (Smith & Nichols, 2009). Statistical significance of these voxel-wise values was then determined via permutation testing. Voxel-wise values were compared to a null distribution of values equally spread around a baseline of zero. Null distribution values were computed over 10,000 iterations by inverting positive and negative signs of the voxel values for a random half of the participants. During each iteration, TFCE values were calculated separately for positive and negative valued voxels, resulting in null distributions for both positive and negative values. The next step involved identifying voxels in which the actual TFCE value was larger than over half of the values generated during the permutations. For these voxels, the TFCE correction procedure took the number of permutations where this was the case, and scaled this to be a value between 0 and 0.5 if the actual TFCE value was negative, or 0.5 and 1 if the actual TFCE value was positive. These values were then computed into a *z*-score using the inverse normal cumulative distribution function. TFCE-corrected maps were voxel-wise thresholded at *z *= 1.96, which corresponds to *p *< .05 after correction for multiple comparisons.

#### 2.2.2 Searchlight mask

We conducted searchlight analyses within a pre-defined binary mask, based on previous work in the identity perception literature. The mask was based on group-level probabilistic maps of face-selective, voice-selective, and multi-modal person-selective regions, based on functional localiser tasks conducted in a separate study by Tsantani et al. (2019). Voice-selective regions were identified by contrasting listeners’ neural responses to human (verbal and non-verbal) vocalisations compared to man-made or environmental sounds in two separate localiser tasks (Belin et al., 2000; Tsantani et al, 2019). These regions included bilateral superior temporal sulci (STS) and superior temporal gyri (STG), which also encompassed bilateral temporal voice areas. Face-selective regions were identified by comparing neural responses to silent non-speaking videos of famous and non-famous faces to silent videos of moving natural or man-made objects. These regions comprised regions within the right occipital gyrus (“occipital face area”/OFA) and the right fusiform gyrus (“fusiform face area”/FFA), as well as the right posterior STG. Multi-modal person-selective regions were established by comparing neural responses to audio-visual speaking clips of famous and non-famous people to audio-visual clips of moving man-made objects or natural scenes. These regions included the precuneus, posterior cingulate, frontal pole, and orbitofrontal cortex.

Prior to our analyses, these areas were combined into a single binarised mask, thus representing a wide range of brain areas associated with identity processing for uni- and multimodal stimuli. This mask was then further thresholded to include voxels present in the individual normalised masks of at least 10 participants (33.3%) from the Tsantani et al. (2019) sample. Finally, the mask was warped, resliced, and resampled into the same MNI space and resolution as participants’ functional data in the NNDb dataset (Aliko et al., 2020). The mask is available on the Open Science Framework: https://osf.io/bh7np.

### 2.3 Results

**Fig. 2.**
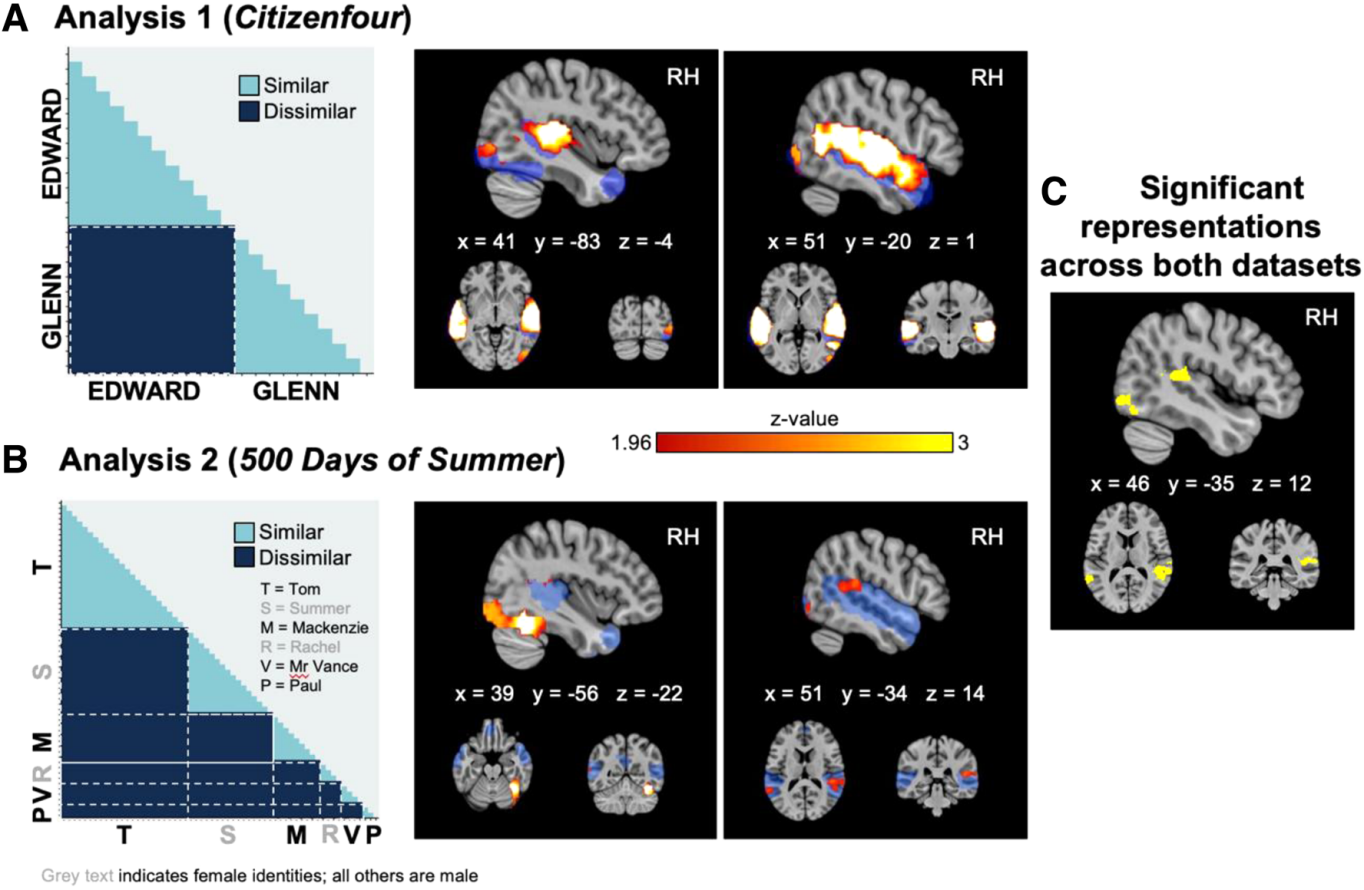
Model representational dissimilarity matrices for identity and group-level searchlight maps for (A) *Citizenfour* and (B) *500 Days of Summer* datasets. Similarity of patterns of neural activity was compared to hypothetical models featuring within- (similar) and between- (dissimilar) identity comparisons. Red-orange clusters show significant correlations following cluster-level TFCE corrections and voxel-wise thresholding at *z *= 1.96. The pre-specified searchlight mask (in which searchlight analyses were conducted) is shown in blue. Supplementary figures show observed neural dissimilarity for within- and between-identity comparisons at peak voxel locations. (C) Unweighted spatial overlap of significant correlations observed across both *Citizenfour* and *500 Days of Summer* datasets. All coordinates are in MNI space.

Figure 2A shows the group-level results of the searchlight analyses, following adjustments for multiple comparisons using TFCE and voxel-wise thresholding at *z *= 1.96. The searchlight maps illustrate areas in which similarities in patterns of neural activity were significantly correlated with predicted similarities in the model RDM based on identity. In other words, patterns of neural activity in these voxels appeared to differentiate between different identities, but show consistencies in response to variable instances of the same identity. Within our pre-defined searchlight mask, the analyses revealed significant correlations in three large clusters, located in the left and right STG/STS, and in the lateral occipital cortex. Cluster statistics are reported in Table 1.

**Table 1.** Group-level searchlight results for Analysis 1 *Citizenfour,* in which similarities of patterns of neural activity were compared to a hypothetical model of two identities

Location	Peak voxel								Cluster statistics		
	Fisher-transformed correlation	SD	*z*-value (max)	x	y	z	Noise ceiling		*z*-value (mean)	SD	Size (mm^3^)
							Lower	Upper			
Left superior temporal gyrus/sulcus	0.074	0.048	3.72	-66	-12	15	0.717	0.751	3.01	0.49	54,621
Right superior temporal gyrus/sulcus	0.074	0.050	3.72	45	-36	15	0.668	0.710	3.07	0.51	55,686
Right inferior occipital gyrus	0.101	0.059	2.82	48	-78	6	0.631	0.678	2.32	0.24	2435

Note. Results show statistically significant clusters exceeding 20 mm^3^, following adjustments for multiple comparisons using TFCE and voxel-wise thresholding at *z *= 1.96. Fisher-transformed correlations refer to correlations between the model RDM (expressing telling-together and telling-apart predictions) and the brain RDM (expressing observed neural dissimilarity). The lower and upper bounds of the noise ceiling (calculated after Nili et al., 2014) indicate the range of Pearson correlation values that could be expected between the best possible model RDM and the brain RDM in each peak voxel.

Clusters of significant correlations were notably widespread within our pre-defined searchlight areas, which raised questions over whether the spatial specificity of detected correlations was localised to established face and voice processing areas, or more distributed across other areas in the brain that were excluded by our mask. To investigate this, we conducted supplementary exploratory whole-brain searchlight analyses. These analyses followed the procedure outlined above, except that we used a whole-brain searchlight mask rather than the theoretically motivated mask based on face-, voice-, and person-processing regions of interest (Tsantani et al., 2019). The whole-brain analyses showed near-identical correlation clusters that were again localised to established face- and voice-sensitive regions, ruling out the possibility that clusters in additional regions were masked by our original approach. Brain maps for exploratory whole-brain analyses can be accessed as supplementary materials on the Open Science Framework (https://osf.io/vu23z/).

Our findings revealed representations of person identity in three distinct brain areas. These included clusters in superior temporal areas that align with areas previously associated with voice identity processing (Belin et al., 2004; Luthra, 2021; Maguinness et al., 2018; Schall et al., 2014; Tsantani et al., 2019; von Kriegstein & Giraud, 2004; von Kriegstein et al., 2003), as well as right posterior STS (rpSTS), which has in previous work shown selectivity for both faces and voices as well as cross-modal identity integration between both modalities (Tsantani et al., 2019). We further found evidence for representations of person identity in the lateral occipital cortex, in areas overlapping with regions defined by Tsantani et al. (2019) as the OFA. The OFA has been previously implicated in generalising identity-related information across variable stimuli of the same person (Tsantani et al., 2019) and different viewpoints of the same face (Anzellotti et al., 2014), suggesting that this region is able to “tell together” multiple variable instances of the same identity.

Overall, our findings provide evidence of a neural basis for distinct representations of person identities based on the face and voice, where patterns of neural activity are more similar in response to instances of the same person compared to instances of two different people. This is a novel demonstration that the brain represents person identity-related information with tolerance to naturalistic within-person variability in the sound and appearance of individuals (“telling together”), in addition to discriminating between identities (“telling apart”). Importantly, despite our study being radically different in design, our findings offer convergent evidence that cortical areas previously associated with identity processing via experimental studies are similarly engaged in representing person identities during naturalistic, dynamic stimulation, and in the absence of any explicit experimental task or manipulation.

## 3 Analysis 2: *500 Days of Summer*

The first analysis demonstrated that brain responses encode specific neural representations for different person identities in response to naturalistic stimuli. We identified distinct clusters where patterns of neural activation were similar for variable instances of the same person, but distinct in response to different people. However, identity comparisons were restricted to a single pair of people, therefore differences in neural responses may have been driven by broader demographic, visual, or auditory differences, rather than by specific identity encoding. For example, neural dissimilarity could be attributed to differences in hair colour or facial accessories (e.g., Edward Snowden has blonde hair and is often seen wearing glasses, while neither characteristic applies to Glenn Greenwald) or coarse-grained acoustic differences in voice characteristics (e.g., mean pitch) that alone would not be diagnostic of these specific identities. The audio-visual presentation of each identity was highly variable (see supplementary materials on the Open Science Framework, https://osf.io/vu23z/, for examples), which provides a protecting factor against this possibility. However, to address this potential limitation, we replicated our analysis on fMRI data from a second independent group of participants, who watched the romantic comedy *500 Days of Summer*. The ensemble cast in this movie facilitated comparisons across a greater number of person identities (six in total), who varied considerably in their demographic characteristics, visual appearance, and voice properties. This second analysis again predicted that neural patterns of activation should still be more similar for response comparisons within the same identity, and that this should be the case for all the identities featured within the model. We simultaneously predicted that neural responses should be dissimilar for comparisons between any given identity and *any other identity*, regardless of similarities in demographic or idiosyncratic characteristics. Thus, this analysis enabled us to probe generalisation of identity representations beyond the characteristics of two specific identities.

### 3.1 Materials and methods

#### 3.1.1 MRI data

We conducted our second analyses on another subset of data from the Naturalistic Neuroimaging Database (Aliko et al., 2020). This subset comprised data from 20 participants who watched *500 Days of Summer* (ten female, aged 19-53 years old, mean age: 27.7 years). Again, participants were right-handed native English speakers, with no known history of neurological disorders, and no hearing or visual impairments. The MRI data were acquired and pre-processed using the same procedures as those outlined for the *Citizenfour* subset.

### 3.2 Experimental design and statistical analysis

#### 3.2.1 Representational dissimilarity matrices

RDMs were constructed in a similar manner to those constructed for the *Citizenfour* dataset in the first analysis. However, for the *500 Days of Summer* dataset, we included six identities. We first identified characters who had substantial dialogue within the movie, defined as having at least 20 utterances, a total speech duration of over 1 minute, and speech in at least three different scenes. Scenes were again labelled based on a change in setting or timepoint in the narrative. These criteria were met for six characters: Tom, Summer, Rachel, McKenzie, Paul, and Mr. Vance. We then identified the scenes in the movie during which these characters spoke. We focused only on utterances that had a duration of at least 2 seconds and in which the character’s face was present throughout. Finally, we only included scenes where the character had a total of at least 4.5 seconds of speech that met the above criteria. This procedure resulted in a final list of identity-scene combinations, which were included as items in the model RDM. The number of included scenes varied for each identity due to the above inclusion criteria and prominence in the film (Tom: 24 items/88 utterances/576 seconds; Summer: 16 items/50 utterances/323 seconds; McKenzie: 9 items/16 utterances/62 seconds; Mr. Vance: 4 items/14 utterances/98 seconds; Rachel: 4 items/10 utterances/72 seconds; Paul: 3 items/9 utterances/53 seconds). Examples of the modelled audio-visual instances of identities can be found in the online supplementary materials on the Open Science Framework (https://osf.io/vu23z/).

As with the previous analysis, only one triangular half of the matrix was included in the analysis, to avoid duplicate comparisons and to exclude comparisons of items with themselves. Same-identity comparisons were coded as similar (0) and different-identity comparisons were coded as dissimilar (1), following the convention of the *Citizenfour* identity RDM in the previous analysis. The model RDM for identities in *500 Days of Summer* is illustrated in Figure 2B.

#### 3.2.2 Representational similarity analysis

Analyses were conducted using the same representational similarity analysis procedure and searchlight mask outlined in the previous analysis. Individual utterances were modelled as events using the same procedure as that used for the *Citizenfour* dataset, and we again constructed contrast estimate maps for each item (speaker-scene combination) versus baseline. The baseline was also modelled in the same manner as the previous analysis, by modelling events in *500 Days of Summer* when there were no visible faces or audible speech (129 events with a total duration of 4 minutes 42 seconds). This resulted in a participant-level measure of the neural responses to audio-visual speech by different characters across different scenes within the movie. We then used the CoSMo MVPA MATLAB toolbox (Oosterhof et al., 2016) to conduct searchlight analyses within the pre-defined mask, again using a spherical searchlight of 100 voxels. We first computed voxel-wise neural dissimilarity by comparing voxel patterns between identity-scene items (using 1 minus Pearson correlation), resulting in an observed RDM for that searchlight. This observed RDM was then correlated with our model RDM using Pearson correlation. This procedure produced voxel-wise Pearson correlation statistics, which then underwent Fisher r-to-*z* transformation. For each participant, this resulted in a brain map of Fisher-transformed Pearson correlation coefficient values for each voxel within the searchlight mask, which expressed how well the patterns of neural activity corresponded with sensitivity to person identity. Following the previous analysis, we conducted group-level one-sample t-tests to determine whether voxel-wise correlations significantly differed from zero. Group-level *z*-scores were again adjusted for multiple-comparisons using TFCE.

### 3.3 Results

Figure 2B shows the group-level results of searchlight analyses in the *500 Days of Summer* dataset, in which we compared neural responses to audio-visual speech uttered by six different characters within the movie. Neural activity was in response to speech during which an individual talker’s face was visible on-screen, and their voice was audible, throughout. As with the previous analysis, significant correlations depict voxels in which observed neural dissimilarity between items aligned with our model RDM of person identity. In these voxels, patterns of neural activity were similar for within-speaker comparisons (characteristic of telling together) and dissimilar for between-speaker comparisons (characteristic of telling apart). Results are again displayed in the pre-defined searchlight mask, following adjustments for multiple comparisons using TFCE and voxel-wise thresholding at *z *= 1.96. The results indicated significant correlations in multiple clusters across bilateral STG/STS, as well as lateral occipital cortex and right fusiform gyrus. Cluster statistics are reported in Table 2.

**Table 2.** Group-level searchlight results for Analysis 2 *500 Days of Summer,* in which similarity of patterns of neural activity were compared to a hypothetical model of dissimilarity for six identities

Location	Peak voxel								Cluster statistics		
	Fisher-transformed correlation	SD	*z*-value (max)	x	y	z	Noise ceiling		*z*-value (mean)	SD	Size (mm^3^)
							Lower	Upper			
Left superior temporal gyrus/sulcus	0.021	0.016	2.32	-63	-51	12	0.578	0.628	2.10	0.09	1065
Right posterior superior temporal sulcus	0.025	0.022	2.44	48	-33	12	0.528	0.587	2.12	0.11	3340
Right superior temporal gyrus	0.020	0.024	2.03	66	-24	9	0.553	0.607	2.00	0.02	117
Right inferior occipital gyrus/ fusiform gyrus	0.030	0.023	3.54	36	-57	-12	0.619	0.663	2.66	0.41	11,523
Right fusiform gyrus	0.014	0.017	2.02	48	-78	6	0.523	0.583	2.01	0.02	21

Note. Results show statistically significant clusters exceeding 20 mm^3^, following adjustments for multiple comparisons using TFCE and voxel-wise thresholding at *z *= 1.96. Fisher-transformed correlations refer to correlations between the model RDM (expressing telling-together and telling-apart predictions) and the brain RDM (expressing observed neural dissimilarity). The lower and upper bounds of the noise ceiling (calculated after Nili et al., 2014) indicate the range of Pearson correlation values that could be expected between the best possible model RDM and the brain RDM in each peak voxel.

We conducted additional exploratory whole-brain analyses to investigate the spatial specificity of our results, following the same procedure outlined in Analysis 1. We again observed that correlated clusters were specifically localised to established face- and voice-selective areas even when a whole-brain analysis was conducted. The brain map results for the exploratory whole-brain analyses can be accessed on the Open Science Framework (https://osf.io/vu23z/).

Using the *500 Days of Summer* dataset, we replicated and extended our findings from the first analysis to identify regions that encode person identity across more than two identities. Convergent with the experimental neuroimaging literature, we found that the neural response patterns within multiple face- and voice-selective processing areas represent person identity, shown by similar patterns of brain activity in response to variable instances of the same identity, and dissimilar patterns of brain activity in response to different identities. There are notable consistencies across the findings from the *Citizenfour* and *500 Days of Summer* datasets (Fig. 2C): For *500 Days of Summer*, we again observed significantly correlated neural patterns of activation with our identity model in bilateral STG/STS, albeit significant correlations were found in an area that is smaller in size and is also more posteriorly located than for *Citizenfour*. As previously outlined, posterior areas within bilateral STS have been associated with phonological processing and analysis of sensory voice features (Luthra, 2021; Maguinness et al., 2018), and also with modality-general person identities (Tsantani et al., 2019). Analysis 2 also revealed widespread sensitivity to identity within the right inferior occipital gyrus, which not only overlapped with a region of the right inferior occipital gyrus identified in Analysis 1, but also extended more anteriorly into fusiform gyrus. Both right occipital and fusiform gyri are widely established as regions that demonstrate sensitivity to face information (Anzellotti et al., 2014; Guntupalli et al., 2017; Rossion et al., 2003; Tsantani et al., 2019). Responses in these regions have recently been shown to be hierarchically organised for identity representation, containing information sufficient to discriminate identities based on image-related (OFA) versus more abstracted (FFA) stimulus properties. The representational content of neural patterns observed in OFA and FFA in response to feature films may be similarly organised: Perceptual data are not available with the NNDb dataset, but future research including the collection of post-hoc perceptual ratings (e.g., physical characteristics and traits of the movie’s characters) may provide insights.

Consistencies across Analysis 1 and Analysis 2 demonstrated replication and generalisability of our findings, as neural representations of person identities were detected in overlapping clusters across two independent groups of participants, and in response to different sets of identities (Fig. 2C). However, there were also some differences regarding where, and to what extent, neural representations of identity were observed in one data set versus the other. On the one hand, this is not surprising: The two analysed movies differ in many respects, from the size and diversity of the cast, to the nature of the dialogue, narrative, and cinematography, which under task-free observation may lead to variations in the salience of face and voice identities across the two films. For example, we might have expected greater topographical consistency of person representations across Analysis 1 and Analysis 2 if the movie datasets had been obtained under conditions in which the number, demographic profile, and weighting (e.g., in terms of onscreen time, spoken lines, and narrative importance) of the main characters were matched. However, such a hypothetical experiment would be barely reflective of how people encounter other identities in the movies, not to mention in everyday life.

Further, discrepancies may also arise from how we have coded identity in our analyses: For the *500 Days of Summer* hypothetical model RDM, between-identity dissimilarity is equivalently high for all identity pairings (e.g., Tom vs. Summer is modelled with equivalent dissimilarity to Tom vs. Mr Vance), while there was only one identity pairing for Analysis 1. A strength of this approach was that correlation clusters in the *500 Days of Summer* analysis should be less likely to reflect neural sensitivities to confounding between-identity dissimilarities other than person identity (i.e., overlaps in general physical and demographic characteristics). The notion that the brain discriminates individual identities in this absolute fashion also aligns with behavioural evidence from face and voice identity sorting studies, in which participants are typically error-free in their ability to tell identities apart but can struggle with “telling together” across naturally varying stimuli (e.g., Jenkins et al., 2011; Lavan, Burston, & Garrido, 2019). However, this binary approach to coding identity is coarse-grained and unlikely to fully reflect how identity are processed. Future experimental work with greater control over (or capacity to estimate) the relative magnitude of physical and perceptual between-identity differences within naturally varying stimuli may be able to test whether—and where—identity is represented in terms of absolute or relative between- and within-person similarity.

Combined, the *Citizenfour* and *500 Days of Summer* analyses provide evidence for neural representations of distinct person identities. These representations may play a key role in providing a stable percept of person identity, which can, in turn, support telling people together and telling them apart in light of variable face and voice information.

## 4 Analysis 3: *Citizenfour*—Delineating Between Face and Voice Identities

The first two analyses characterised neural representations of person identity in response to naturalistic audio-visual stimuli, from which participants could simultaneously view a person’s face and hear their voice. From these analyses alone, we do not have any insights into the extent to which representations were driven by the two different modalities. Therefore, the aim of the third analysis was to provide a more nuanced characterisation of neural representations associated with person identity, by investigating whether various areas engaged in identity encoding were preferentially sensitive to either face or voice identity information. This final analysis was designed as a first step in teasing apart the preferential encoding of face versus voice information in identity representations, by comparing neural responses to speech samples in which either only face or only voice identity information is shared.

### 4.1 Materials and methods

#### 4.1.1 MRI data

Our third set of analyses were conducted on the *Citizenfour* subset of the Naturalistic Neuroimaging Database (Aliko et al., 2020). This dataset was selected because the movie featured substantial intervals of dialogue in which the face on-screen did not match the voice of a different person off-screen, which was simultaneously heard in the soundtrack.

### 4.2 Experimental design and statistical analysis

#### 4.2.1 Representational similarity analysis

The purpose of this analysis was to delineate between sensitivity to identity based on voice information, and sensitivity to identity based on face information. This required us to compare neural responses to stimuli for which only the face or only the voice belonged to the same identity. We again focused on instances of speech and on-screen appearances by Edward Snowden and Glenn Greenwald as the two most prominent identities within *Citizenfour*. We aimed to compare neural responses to stimuli in which only the face identity or the voice identity was consistent, in order to delineate between representations that were preferentially sensitive to face or voice identity information. As participants were naturalistically exposed to face and voice information by watching feature-length films, there were not enough instances where participants were presented with a face in the absence of any voice (or vice versa) to ensure a sufficiently powered analysis. Instead, we isolated alternative events in the movie during which the face of one person was visible on-screen while another person was heard talking off-screen. These instances of speech are henceforth described as incongruent, whereas instances of speech in which the face on-screen belonged to the same identity as the person talking are referred to as congruent (i.e., the events of interest used in the first analysis).

We isolated incongruent utterances that lasted a duration of 2 seconds or more, and only included utterances from scenes which featured a total duration of at least 10 seconds for a particular incongruent combination (either *EDWARD FACE + GLENN VOICE [EF+GV] or GLENN FACE + EDWARD VOICE [GF+EV]*). This criterion resulted in a shortlist of scenes for each incongruent combination, which were then selected as items for our analysis. In addition, we included a subset of items from the first analysis, which reflect congruent instances of speech in which the same person’s face was on-screen (either *EDWARD FACE + EDWARD VOICE [EF+EV] or GLENN FACE + GLENN VOICE [GF+GV])*. This congruent subset included scenes in which the person’s cumulative speech duration was 10 seconds or more, in order to match the selection criterion for incongruent items. This resulted in five scenes for each congruent and incongruent combination (*EF+EV, EF+GV, GF+EV, GF+GV*), resulting in 20 items in total. As with the previous two analyses, we modelled participants’ neural responses to timestamped speech events that were included in each of the congruent and incongruent items. Events for each item (face-voice-scene combination—e.g., *GF+EV 1*) were modelled as separate contrast estimates, modelled against baseline. The baseline was modelled on the same events as Analysis 1, based on events in the film during which no visible face or audio voice was present.

**Fig. 3.**
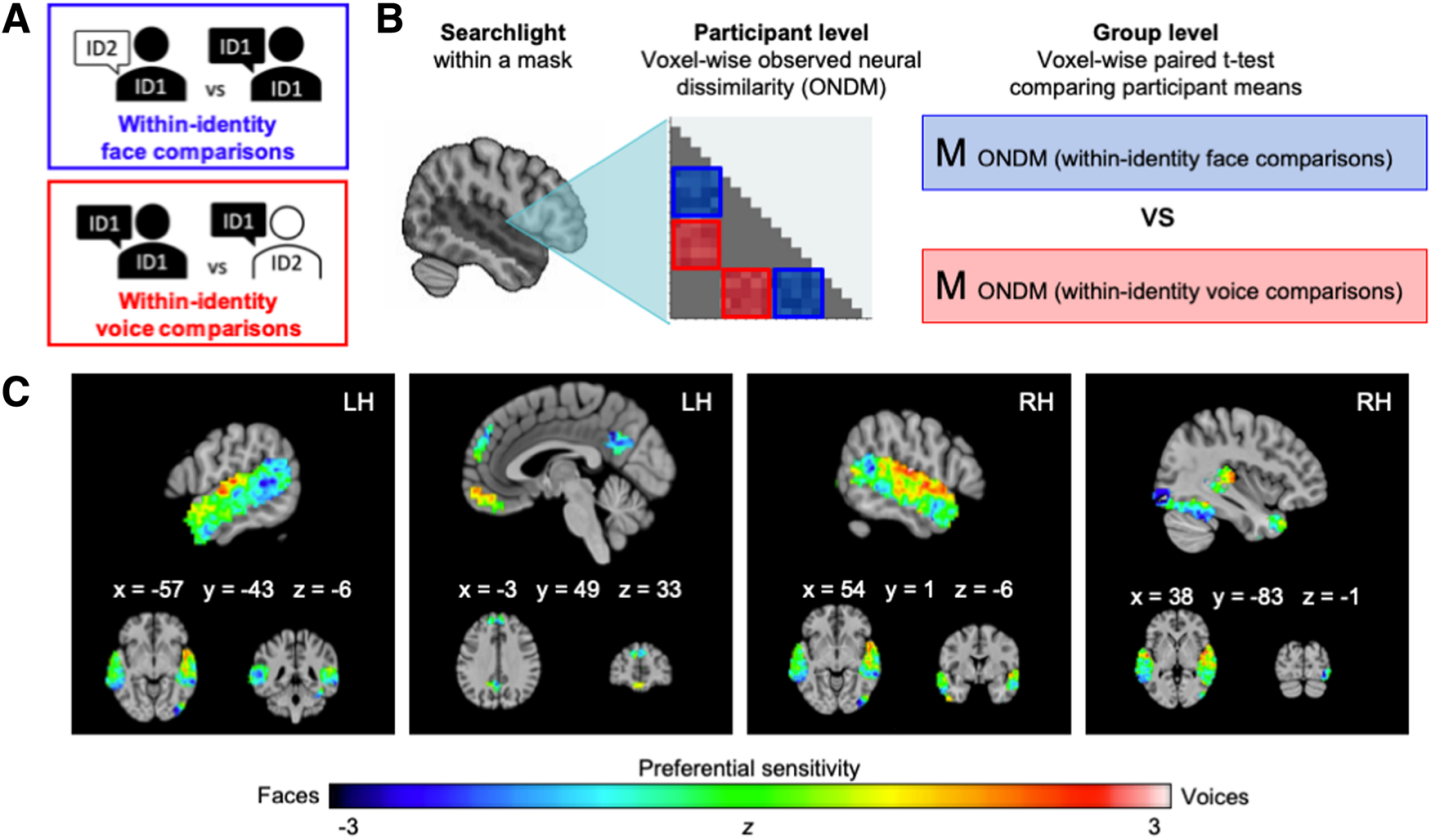
(A) Illustration of congruent and incongruent identity items. (B) Illustration of representational similarity analysis procedure. (C) Uncorrected and unthresholded *z*-map from a paired samples t-test comparing voxel-wise observed neural dissimilarity between within-identity face comparisons and within-identity voice comparisons. Negative values indicate clusters with preferential sensitivity for face identities, as voxel-wise observed neural dissimilarity was lower for shared-face comparisons compared with shared-voice comparisons. In contrast, positive values denote preferential sensitivity to voices. All coordinates are in MNI space.

In this analysis, we took an alternative representational similarity approach, by directly extracting and comparing observed neural dissimilarity arising from comparisons with shared face identities and comparisons with shared voice identities. This analysis differed from the previous analyses as we did not construct an *a priori* model RDM, instead we directly compared observed neural dissimilarity across comparisons of events during which only face or voice identity information was shared. We initially followed the same searchlight procedure as the previous two analyses to create neural dissimilarity matrices. For each participant, a spherical searchlight extracted neural response patterns for each item at each voxel within the searchlight mask, based on patterns of activity in the surrounding 100 voxels. We then calculated voxel-wise observed neural dissimilarity between items by comparing the neural response pattern across each item using Pearson correlations, which were subtracted from one to express dissimilarity. We excluded comparisons between two congruent items, or between two incongruent items, as in these comparisons both face and voice identity, or neither, would be shared. These exclusions thus enabled comparisons across items that shared identity in one modality only (Fig. 3A). This resulted in an observed neural dissimilarity matrix for each voxel location, which demonstrated how similar neural patterns of activation were in response to naturalistic stimuli in which only the same face or the same voice was present.

At this stage, our analysis departed from the procedure used in previous analyses. For each participant, we separately calculated mean observed neural dissimilarity for all same-face comparisons and all same-voice comparisons at each voxel within the mask (Fig. 3B). This resulted in two Pearson correlation statistics for each voxel in the ROI mask for each participant: One expressed observed neural dissimilarity based on shared face information, and the other expressed observed neural dissimilarity based on shared voice information. Finally, we conducted a group-level pairwise t-test to detect voxels in which neural dissimilarity was statistically lower in response to shared information for one modality compared to another. This enabled us to probe neural areas that were relatively more sensitive to face identity information compared to voice identity information (and vice versa).

### 4.3 Results

The group-level results revealed distinct areas showing preferential sensitivity to face or voice identities, shown as an uncorrected *z*-map in Figure 3C. Table 3 reports clusters that demonstrated statistically greater sensitivity to voice identities over and above face identities (and vice versa) when thresholded at voxel level (*z *<= -1.96 for preferential sensitivity to faces and *z *>= 1.96 for preferential sensitivity to voices). However, these findings did not remain significant after applying cluster-level TFCE corrections.

**Table 3.** Group-level searchlight results for Analysis 3 *Citizenfour,* reporting clusters that demonstrated statistically greater sensitivity to face identities compared to face identities (and vice versa) when thresholded at voxel level

Modality	Location	Peak voxel						Cluster statistics		
		1—Pearson Correlation	SD	*z*-value	x	y	z	Mean *z*-value	SD	Size
Face	Left superior temporal gyrus/sulcus	0.599	0.168	-3.22	-63	-48	15	-2.18	0.20	508
		0.667	0.137	-2.59	-66	-51	-3	-2.11	0.13	156
	Right inferior occipital gyrus	0.843	0.040	-3.76	36	-93	-15	-2.65	0.38	1621
	Right fusiform gyrus	0.821	0.080	-3.25	39	-51	-30	-2.29	0.25	653
	Right superior temporal gyrus/sulcus	0.801	0.124	-3.29	63	-39	-12	-2.22	0.19	213
		0.681	0.137	-2.66	60	-15	-15	-2.16	0.14	195
	Precuneus/posterior cingulate cortex	0.917	0.051	-3.14	-6	-57	27	-2.30	0.28	168
		0.908	0.065	-3.13	-3	-54	33	-2.34	0.30	88
Voice	Left superior temporal gyrus/sulcus	0.548	0.102	3.34	-54	-12	3	2.29	0.28	193
		0.576	0.120	2.92	-54	-18	9	2.21	0.20	166
	Right posterior superior temporal sulcus	0.489	0.134	3.43	57	-18	12	2.33	0.32	833
	Right superior temporal gyrus	0.331	0.163	3.28	51	-6	-9	2.22	0.25	216
		0.468	0.150	2.97	51	-12	6	2.17	0.19	99
		0.313	0.115	2.63	42	-21	9	2.14	0.14	87
	Orbitofrontal cortex	0.236	0.125	2.68	51	18	-12	2.17	0.17	55

Note. Results show statistically significant clusters exceeding 20 mm^3^, voxel-wise thresholded above *z *= 1.96 and below *z* = -1.96. Clusters did not survive these voxel-wise *z*-value thresholds when *z*-values were corrected for multiple comparisons under TFCE. One minus Pearson correlations reflect average dissimilarity in neural responses for telling-together comparisons, in which identity is shared across either face or voice modalities.

Results corrected for voxel-wise multiple comparisons revealed that observed neural dissimilarity for comparisons with shared-face identities was significantly lower than for comparisons with shared-voice identities in right inferior occipital and fusiform gyri, as well as clusters distributed across bilateral STG/S, precuneus, and posterior cingulate cortex. As lower observed neural dissimilarity indicates that response patterns are more similar, these significant differences are interpreted as preferential sensitivity for face identity information over voice identity information. In contrast, preferential sensitivity for voice identity information (measured by significantly lower observed neural dissimilarity for same-voice comparisons compared to same-face comparisons) was observed in separate clusters within bilateral STG/S, including the right posterior STS. In addition, we detected a small cluster that demonstrated preferential sensitivity to voice identities over face identities within the orbitofrontal cortex.

The aim of this final analysis was to dissociate contributions of face versus voice information to neural representations of identity. The results revealed multiple clusters in which neural representations of identity were better characterised by shared identity information across one modality relative to another, although these differences were only significant when voxel-level thresholded, and did not survive cluster-level correction for multiple comparisons. These clusters corresponded reliably with previous neuroimaging evidence, as preferential sensitivity for face identities was mostly localised to established face-selective cortical areas, including the right occipital and fusiform gyri (Nestor et al., 2011; Pitcher et al., 2011; Rossion, 2008; Rotshtein et al., 2005, 2007; Tsantani et al., 2019, 2021). Similarly, voice-preferential clusters were identified in well-documented voice-selective areas across bilateral STG/S (Andics et al., 2010; Belin & Zatorre, 2003; Belin et al., 2004; Luthra, 2021; Maguinness et al., 2018; Schall et al., 2014; von Kriegstein & Giraud, 2004; von Kriegstein et al., 2003, 2010; Zäske et al., 2017). We also observed preferential sensitivity to one modality over the other in multimodal person-selective areas, including the right posterior STS, precuneus, and orbitofrontal cortex (Anzellotti & Caramazza, 2017; Campanella & Belin, 2007; Deen et al., 2015; Davies-Thompson et al., 2019; Hasan et al., 2016; Watson, Latinus, Charest, et al., 2014; Watson, Latinus, Noguchi, et al., 2014; Tsantani et al., 2019). Descriptively speaking, preferential sensitivity to face identities appeared more topographically widespread than preferential sensitivity to voice identities. This observation aligns with reports from the behavioural literature suggesting a dominance of the visual modality for identity perception, where identity perception from faces is generally both more accurate and robust than identity perception from voices (Barsics, 2014; Stevenage & Neil, 2014).

## 5 General Discussion

In this study, we investigated neural representations of person identity in naturalistic settings, by analysing fMRI data of participants watching feature-length films, and comparing neural activation patterns in response to audio-visual presentations of people that appeared in the films. We found distinct neural representations for individual identities in several areas previously associated with face, voice, and person perception, shown by similarities in neural response patterns to variable presentations of the same identity (“telling together”) and dissimilarities in neural response patterns to instances of different identities (“telling apart”). Evidence for neural representations of audio-visual identities was replicated across two independent datasets, establishing the degree of generalisability of the finding beyond a specific set of identities. Finally, we modelled instances of mismatching face and voice identities to test for preferential representation of either face-only or voice-only identity information, thus further probing the content of representations. Several sites of preference were identified for each modality, with more topographically widespread evidence of preferential sensitivity for face identity information.

This study is one of the first to demonstrate how the brain represents person identity information from ecologically valid, naturally varying stimulation in the absence of an experimental task. It builds on previous work that has demonstrated that naturalistic stimulation from movies can increase engagement, result in stronger and more widespread brain activation, and reveal different functional networks of activity (Aliko et al., 2020; Haxby et al., 2020; Jiahiu et al., 2020). The identity-sensitive regions identified in the current findings align closely with existing knowledge about face and voice processing, and therefore complement, replicate, and validate previous work by showing that similar brain areas are engaged in representing identities under both task-based and naturalistic exposure to people. Examining the neural representations that arise during naturalistic identity perception also provides a new dimension of evidence, as additional contextual information yields greater opportunities for semantic and associative memories to play a role. Such a role may have been underspecified in traditional experimental designs where context, familiarity, and other potential sources of information are often minimised or entirely removed. Not only does this compromise ecological validity, but it also limits the important aspect of the real-world context proposed by leading theoretical models of face and voice perception. For example, Bruce and Young (1986) propose that identification is supported by stored biographical information about known individuals, and also the wider cognitive system, which supports identity perception by recalling associative memories. Bruce and Young (1986) further suggest that the wider cognitive system directs attention toward different functional components of the face processing system to suit the perceptual task at hand (e.g., picking out a familiar face from a crowd; discriminating between two unfamiliar identities). Therefore, the rich naturalistic contexts incorporated into our study design provided participants with the opportunity to engage with the same knowledge and perceptual tasks that theoretical models propose they use spontaneously in real-life situations. This also means that, although the topographical locations of identity representations strongly align with previously defined face- and voice-selective cortical regions, the content of the observed person identity representations may extend beyond face and voice identity information to incorporate additional higher-order knowledge (e.g., demographic profile, (perceived) character traits, biography/narrative role; see Tsantani et al., 2021).

Beyond removing experimental constraints, our study is further novel in that it capitalises on the fact that feature films include multiple variable instances of the same person, making it one of the first to comprehensively model identity perception by incorporating both within- and between-person comparisons. The need to model within-person variability has received attention in recent behavioural work as, in order to recognise people in everyday life, an observer must be able to not only “tell apart” different identities, but also “tell together” multiple instances of the same person into a single perceived identity (Burton, 2013; Lavan, Burston, & Garrido, 2019; Lavan, Burton, et al., 2019). However, this aspect of person identification is routinely underspecified in studies that investigate how person identities are represented within the brain. By combining naturalistic audio-visual stimuli with representational similarity analysis, we were thus able to incorporate these recent conceptual developments in the person perception behavioural literature to a neuroscientific investigation of identity perception in the brain. Our approach has demonstrated how representational similarity can be harnessed as a powerful tool for investigating the neural basis of telling together and telling apart, and offers future potential for investigating more nuanced predictions. The model representational dissimilarity matrices implemented in the current work were fairly reductionist in nature, as predictions assumed that neural responses to the same identity would be equally similar to each other, and that neural responses to different identities would be equally dissimilar to each other. This was essential to our research aim, which was to detect neural representations sensitive to identity independently of any other shared qualities. The success of this initial investigation provides scope to test more fine-grained characterizations of telling together and telling apart in future applications.

As the first of its kind to model representations of person identity under naturalistic and task-free stimulation, the current study offers a starting point for future work to more comprehensively explore neural representations of other people. For example, there is currently very little work tracking how initially unfamiliar identities become familiar in naturalistic settings. Behavioural evidence has shown that familiarity dramatically improves perceivers’ abilities to identify a person from naturally varying stimuli (Burton et al., 2016; Hancock et al., 2000; Jenkins et al., 2011; Kanber et al., 2021; Lavan, Burston, & Garrido, 2019; Lavan, Burton, et al., 2019; Zhou & Mondloch, 2016). Future applications of representational similarity analysis could investigate how neural representations of identity are initially established when we encounter a person for the first time and how these representations may change in their nature and/or content over a longer period of naturalistic exposure. A recent series of electroencephalogram (EEG) studies used representational similarity analysis to investigate the effects of familiarity on face identification, and demonstrated that neural representations differ based on the mode of familiarity (i.e., whether a person is known personally through real-life contact, through media exposure or via brief lab-trained exposure; Ambrus et al., 2021). Thus, familiarity may impact not only the robustness of neural identity representations, but also the way in which they are represented. The current work focused on naturalistic media-based exposure to famous individuals (*Citizenfour*) and fictional movie characters (*500 Days of Summer*). Further investigation may reveal differences between representations of person identity based on the nature of exposure. There is also scope to investigate how identity-based knowledge interacts with other types of processing, such as the extraction of linguistic information. Familiarity has been shown to benefit speech intelligibility (Holmes et al., 2018; Johnsrude et al., 2013; Kreitewolf et al., 2017; Nygaard & Pisoni, 1998; Nygaard et al., 1994), and recent work has investigated the neural basis of these effects by uncovering neural representations that correlate with speech intelligibility benefits from familiar talkers (Holmes & Johnsrude, 2021). Naturalistic exposure provides additional unexplored opportunities to answer these questions, as it allows researchers to better characterise how familiarity shapes neural representations over longer timeframes, with greater within-person variability than most experimental paradigms incorporate.

Future work could also use representational similarity analysis to investigate a more graded depiction of telling apart. While we expect individuals to hold distinct representations for different identities, we may not necessarily expect these representations to be equally distanced in their dissimilarity to each other. Recent studies have used representational similarity analysis to show how dissimilarities between neural representations of different face identities align with differences in visual appearance, gender, and social traits in different face-selective regions. For example, Tsantani et al. (2021) investigated how identity-related information is represented in face-selective regions. Their findings suggested that OFA discriminated identities based on low-level image-based properties, while FFA discriminated identities based on higher order characteristics, such as the perceived similarity between identities, as well as the identities’ perceived traits and gender. These insights could be used to inform more fine-grained depictions of how different identities are represented in relation to one another.

Other work has used representational similarity analysis to identify brain regions in which dissimilarities in neural responses to different individuals are correlated with their proximity to each other in social networks (Parkinson et al., 2017). Naturalistic stimulation via feature-length movies provides a prime opportunity to explore these findings further, as it not only increases ecological validity through varied and extensive exposure to face and voice identities, but it also further embeds them in rich contexts. Consequently, identities are observed communicating with other people and interacting with their environment, as part of a coherent narrative. This rich contextual information can potentially enable researchers to map whether and how social relationships between the observer and an identity or between different identities are represented (Todorov et al., 2006). This could include investigation of how neural representations of identities are shaped by their social interactions (Burr & Dick, 2017), status (Betancourt et al., 2018), or affinity to in-groups and out-groups (Cikara et al., 2011).

To conclude, our study is one of the first to investigate the neural representations that underpin identity perception in naturalistic, task-free settings with dynamic, multimodal stimulation. Our novel application of representational similarity analysis to neural responses to feature-length movies enabled us to characterise how the brain represents information about other people in the real world. Analyses revealed person-identity representations that generalise across naturalistically varying exposures of the same person in areas of right inferior occipital and fusiform gyri, as well as distributed areas across bilateral superior temporal gyri and sulci, including the right posterior superior temporal sulcus. These representations were detected and replicated in overlapping areas across two independent groups of participants in response to different sets of identities. Further analyses revealed preferential representation of either face or voice identity information in associated face- and voice-processing areas, with wider evidence of areas preferring visual identity information. Future work can harness the ecologically valid variation and rich context offered by naturalistic stimuli to better understand how identity information is represented in the brain. This could include investigations into how representations change as familiarity with a person increases, or how contextual world knowledge shapes identity encoding.

## Data Availability

This study was conducted on open-access MRI datasets from the Naturalistic Neuroimaging Database (Aliko et al., 2020). The MRI datasets are available from the OpenNeuro platform at https://doi.org/10.18112/openneuro.ds002837.v1.1.1 (dataset accession number ds002837). The scripts and materials for the analyses reported in this manuscript are openly available on the Open Science Framework (https://osf.io/vu23z/).

## Supplementary Materials

Supplementary material for this article is available here: https://osf.io/vu23z/.
